
JOURNAL INFORMATION
==============================
NLM Title Abbreviation: ScientificWorldJournal
Journal ID: ScientificWorldJournal
NLM Journal ID: 101131163
Journal ID: TSWJ

The Scientific World Journal

ISSN: 2356-6140
EISSN: 1537-744X
Publisher: Wiley

ARTICLE INFORMATION
==============================
PMCID: PMC6084242
PMID: 30147560
DOI: 10.1100/tsw.2001.196
Article ID: 651250
Article version: 1
Subjects: Short Report

Signaling through the Death Receptor CD95 (APO-1/FAS)

Peter Marcus E. *
Ben May Institute for Cancer Research, University of Chicago, USA
*Marcus E. Peter: mpeter@ben-may.bsd.uchicago.edu
Publication date: 2001
Electronic publication date: 2001 Dec 1
Volume: 1
First page: 90
Last page: 90
Copyright: Copyright © 2001 Marcus E. Peter.
Copyright year: 2001
License: This is an open access article distributed under the Creative Commons Attribution License, which permits unrestricted use, distribution, and reproduction in any medium, provided the original work is properly cited.
License URL: https://creativecommons.org/licenses/by/3.0/

==============================
Miami *Nature Biotechnology* Short Reports
*TheScientificWorld* (2001) 1 (S3), 90SR
ISSN 1532-2246; DOI 10.1100/tsw.2001.196

### SIGNALING THROUGH THE DEATH RECEPTOR CD95 (APO-1/FAS)

Marcus E. Peter

Ben May Institute for Cancer Research, University of Chicago
MPeter@ben-may.bsd.uchicago.edu.

**INTRODUCTION AND METHOD.** Using classical immunoprecipitation protocols in combination with high-resolution 2D gels, we identified a complex of proteins that formed around the death domain of the stimulated death receptor CD95 (APO-1/Fas) receptor which we called the death-inducing signaling complex (DISC)(1). We went on to characterize the three major components of the DISC: First, the adaptor FADD/Mort1 was identified (1) then caspase-8 and the caspase-8-like apoptosis inhibitor c-FLIP were cloned (2,3). Formation of the DISC upon apoptosis induction results in activation of caspase-8 by proteolytical cleavage into the prodomain containing two death effector domains (DED) and two active subunits, p18 and p10 (4). Thus, caspase-8 plays an essential role in the execution of death receptor-mediated apoptosis. To determine the localization of endogenous caspase-8 we used a panel of subunit-specific anti-caspase-8 monoclonal antibodies in confocal immunofluorescence microscopy.

**RESULTS.** In human breast carcinoma cells MCF7 caspase-8 predominantly colocalized with and bound to mitochondria. After induction of apoptosis through CD95 or TNF receptor I, active caspase-8 translocated to plectin, a major crosslinking protein (cytolinker) of the three main cytoplasmic filament systems. Plectin was quantitatively cleaved early by caspase-8 in all cells tested during death receptor and drug induced apoptosis. In primary fibroblasts from plectin deficient mice apoptosis induced reorganization of the actin cytoskeleton, as seen in wild type cells, was severely impaired suggesting that during apoptosis plectin is required for the reorganization of the microfilament system (5). Mitochondria play an important role in many form of cell death. All of the apoptogenic activities of mitochondria can be blocked by overexpression of Bcl-2 or Bcl-$x_L$. In certain cell types most of caspase-8 is activated downstream of mitochondria (6). In these Type II cells overexpression of Bcl-2 or Bcl-$x_L$ can inhibit apoptosis, and cleavage of caspase-3 and 8. In Type I cells, however, caspase-8 is mainly activated by the DISC and seems to directly activate caspase-3. CD95-mediated apoptosis in Type I cells cannot be inhibited by overexpression of Bcl-2 or Bcl-$x_L$. Thus, we have identified two independent cell types that either do or do not use mitochondria for execution of apoptosis.

**DISCUSSION.** We have shown that human peripheral T cells that are resistant to CD95 mediated apoptosis are Type II cells which differentiate into apoptosis sensitive Type I cells after prolonged incubation with interleukin-2. Recently, we have focused at a novel function of mitochondria during CD95 mediated apoptosis. We found that expression of Bcl-2/Bcl-$x_L$ gives mitochondria the activity to sequester active caspase-8 thus preventing the execution of apoptosis despite activation of caspase-8 at the DISC. The data suggest that active caspase-8 binds to the mitochondrial protein BAR (7) on the surface of mitochondria. Thus, we have identified a novel mechanism of regulation of caspase-8 activity that might be relevant for regulation of apoptosis and cell proliferation.

**REFERENCES.**

1. Kischkel, F.C., Hellbardt, S., Behrmann, I., Germer, M., Pawlita, M., Krammer, P.H., and Peter, M.E. (1995) EMBO J. 14, 5579-5588
2. Muzio, M., Chinnaiyan, A.M., Kischkel, F.C., O' Rourke, K., Shevchenko, A., Scaffidi, C., Zhang, M., Ni, J., Gentz, R., Mann, M., Krammer, P.H., Peter, M.E., and Dixit, V.M. (1996) Cell 85, 817-827
3. Scaffidi, C., Schmitz, I., Krammer, P.H., and Peter, M.E. (1999) J. Biol. Chem. 274, 1541-1548
4. Medema, J.P., Scaffidi, C., Kischkel, F.C., Shevchenko, A., Mann, M., Krammer, P.H., and Peter, M.E. (1997) EMBO J. 16, 2794-2804
5. Stegh, A.H., Herrmann, H., Lampel, S., Weisenberger, D., Andrä, K., Seper, M., Wiche, G., Krammer, P.H., and Peter, M.E. (2000) Mol. Cell. Biol. 20, 5665-5679
6. Scaffidi, C., Fulda, S., Srinivasan, A., Li, Feng, Friesen, C., Tomasseli, K.J., Debatin, K.M., Krammer, P.H., and Peter, M.E. (1998) EMBO J. 17, 1675-1687
7. Zhang, H., Qunli, X., Krajewski, S., Krajewska, M., Xie, Z., Fuess, S., Kitada, S., Pawowski, K., Godzik, A., and Reed, J.C. (2000) Proc. Natl. Acad. Sci. U S A 97, 2597-2602
